# Reconstructive Methodology in the Synthesis of 2-Aminopurine

**DOI:** 10.3390/molecules29010134

**Published:** 2023-12-25

**Authors:** Artyom O. Neymash, Evgeny N. Ulomsky, Victor V. Fedotov, Semen V. Aminov, Daniil N. Lyapustin, Evgeny B. Gorbunov, Vladislav A. Ishimnikov, Pavel A. Slepukhin, Vladimir L. Rusinov

**Affiliations:** 1 Chemical Engineering Institute, Ural Federal University, 19 Mira St., 620002 Yekaterinburg, Russia; fedotov.viktor@urfu.ru (V.V.F.); aminov.sema@gmail.com (S.V.A.); lyapustin.danil@yandex.ru (D.N.L.); smart23070250@gmail.com (V.A.I.); v.l.rusinov@urfu.ru (V.L.R.); 2 Institute of Organic Synthesis, Ural Branch of the Russian Academy of Science, Sofii Kovalevskoy St. 22, 620137 Yekaterinburg, Russia; nitro@ios.uran.ru (E.B.G.); slepukhin@ios.uran.ru (P.A.S.)

**Keywords:** tetrazololopyrimidines, purines, reduction, nitro group transformations, condensation, reconstructive methodology

## Abstract

A fundamentally new synthetic approach to the synthesis of 2-aminopurine has been developed. It consists in the combination of the creation of a condensed polyazotic heterocyclic tetrazolopyrimidine structure, its transformation into triaminopyrimidine, and its subsequent cyclization into 2-aminopurine. The structure of the obtained compounds was established based on spectral characteristics, and the structure of the intermediate compound **5** was established directly by X-ray diffraction analysis.

## 1. Introduction

The compound 2-aminopurine, as a structural analog of guanine, represents a significant group of chemical materials—products of small molecules that are constructed based on modifications of biologically relevant natural compounds [1]. These molecules include purines and their derivatives, such as nucleosides and nucleotides [2]. The methods of structural modifications of purines and their derivatives are different, including the construction of purines and their glycosidic and/or nucleotide components. Initial studies have been limited to one of three structural modification options: heterocycle, glycoside, and phosphonate. All three techniques exhibited significant efficacy in terms of introducing innovative qualitative approaches for the development of antiviral and antitumor agents to combat-related diseases and infections.

The compound 2-aminopurine and its derivatives represent an important structural element for the creation of antagonists of nucleic acid monomers and their precursors. In addition, it is an intermediate in the biochemical pathways of purine and nucleic acid metabolism. It is also used as a marker to determine the activity of some enzymes involved in these processes [3].

**Figure 1:**
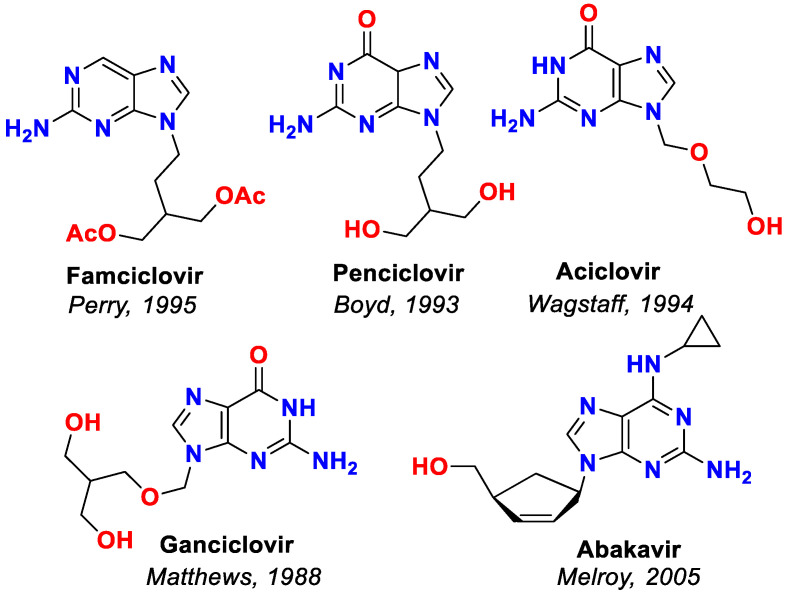
Aminopurine-based medicines [4,5,6,7,8].

The most significant representatives of this group of antiviral drugs are 2-aminopurines and non-natural nucleosides based on them (Figure 1).

Famciclovir is an antiviral medicine used to treat genital herpes and shingles. It works by preventing the viruses that cause these diseases from multiplying [4]. Penciclovir is an antiviral medication used to treat infections caused by the herpes simplex virus. It works by preventing the virus from multiplying and can be used to treat both genital and oral herpes infections [5]. Acyclovir is an antiviral medicine used to treat infections caused by the herpes simplex virus, such as herpes on the lips and genitals. It works by blocking the reproduction of the virus [6]. Ganciclovir is a medicine used to treat viruses such as cytomegalovirus and herpes simplex virus. It works by stopping viruses from multiplying in the body [7]. Abacavir is a medicine used to treat HIV infection. It works by blocking the activity of an enzyme that is needed for the virus to multiply [8]. 

**Scheme 1:**
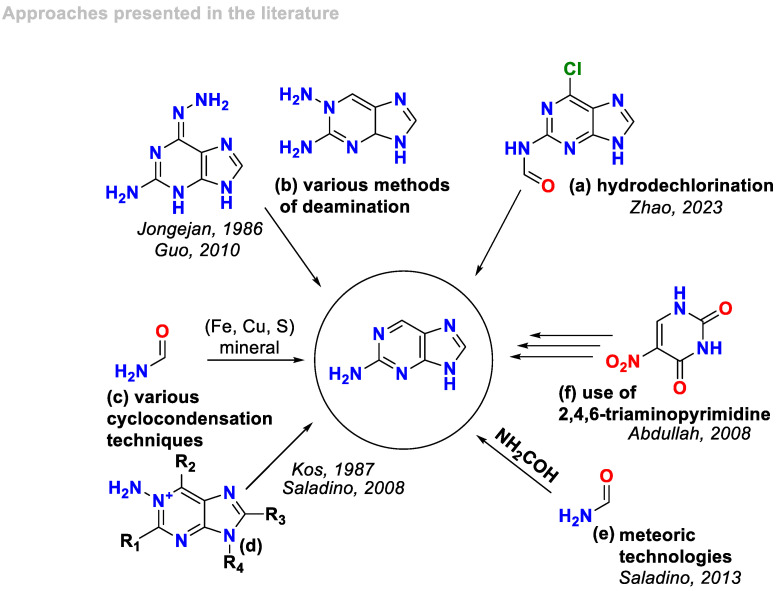
Literary approaches to 2-aminopurine [9,10,11,12,13,14,15].

Various methods for the preparation of 2-aminopurine are presented in the literature (Scheme 1). One of the methods for obtaining the target product is the hydrogenation of 6-chloraminopurine [9]. In other publications [10,11], the authors use deamination methods. Thus, in [10], the dehalogenation of 6-chloropurine by microwave irradiation in water is used. In another study [11], the intermediate product is hydrazinopurine, which is formed by the deamination of 4,9-dihydro-purine-1,2-diamine. In this way, these two methods, which are described above, involve the removal of an amino group from the precursor compound to give 2-aminopurine. Alternatively, various cyclocondensation procedures have been reported in publications [12,13] to obtain the desired product. These methods involve the formation of a cyclic compound by the condensation of suitable precursors. The authors describe the one-pot synthesis of a large panel of nucleic bases and related compounds from formamide in the presence of iron-sulfur and iron-copper sulfur minerals as catalysts. Another paper reports the catalytic effect of several types of meteorites: iron, stony-iron, chondrites, and achondrites [14]. Thus, meteorite technologies are considered as a potential method of synthesis of 2-aminopurine. These technologies involve the use of meteoritic materials or processes to facilitate the synthesis. Another approach to obtain the target product is the application of 2,4,6-triaminopyrimidine via the cyclization of the vicinal diamine group, with 5-nitrouracil as the starting reagent [15]. 

It is important to note that these methods represent some of the recognized approaches to the preparation of 2-aminopurine, and additional methods or variations of them may be found in the scientific literature. The aim of this work was to develop a simple synthetic approach that would reduce the number of steps so that it would not be difficult to obtain the target product.

In the present work, we propose a new method for the preparation of 2-aminopurine based on 2,4,6-triaminopyrimidine involving aminotetrazole and nitropyrimidine as key reagents. This approach is an alternative to the existing methods: aminoformylation and chlorodeoxygenation, which involve the use of phosphoryl chloride and subsequent hydrodechlorinatin. The last two steps are inconvenient in the synthetic application. The method developed by us is devoid of the described disadvantages. It should be noted that the target product is an important object for research and further transformations.

It is important to note that these methods represent some of the recognized approaches for the preparation of 2-aminopurine, and there may be additional methods or variations available in the scientific literature.

In the present work, we propose a new method for the preparation of 2-aminopurine based on 2,4,6-triaminopyrimidine involving aminotetrazole and nitropyrimidine as key reagents.

## 2. Results

### 2.1. Synthesis

As a novel approach for the preparation of purine systems, we propose a synthetic strategy based on the synthesis of tetrazolopyrimidines.

**Scheme 2:**
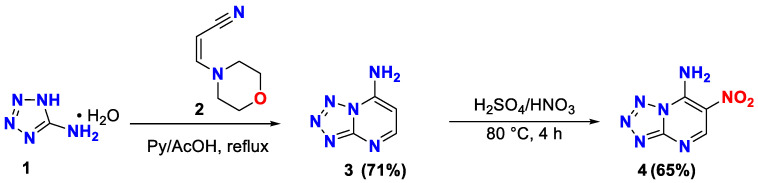
Preparation of 6-nitrotetrazolo[1,5-*a*]-pyrimidine-7 amine **4**.

The interaction of 1H-tetrazol-5-amine **1** with morpholinoacrylonitrile **2** in a 1:1 mixture of pyridine and acetic acid leads to tetrazolo[1,5-*a*]pyrimidine-7-amine 3 (71%) (Scheme 2). Acetic acid and pyridine act as analogs of “ionic liquids”, with the basic properties of pyridine and the acidic properties of acetic acid also favoring condensation reactions [16]. The structure of the obtained tetrazolo[1,5-*a*]pyrimidine-7-amine **3** was confirmed by ^1^H NMR, ^13^C NMR, IR spectroscopy, and elemental analysis data. It should be noted that in the ^1^H NMR spectrum, there are two characteristic doublets with a spin–spin interaction constant of J = 7.5 Hz in the region of δ = 6.59–6.61 ppm and δ = 8.89–8.91 ppm, corresponding to the aromatic protons of the pyrimidine cycle. In turn, the protons of the primary amino group appear as a broadened singlet in the region of δ = 7.74 ppm. The presence of the primary amino group is also confirmed by infrared spectroscopy, as the corresponding spectra show a broadened band of low intensity in the region of 3100 cm^−1^ (Figures S1 and S5, see Supplementary Materials). The second step of the synthesis was the nitration of tetrazolo[1,5-*a*]pyrimidine-7-amine **3**. The choice of a nitrating mixture with a molar ratio of 1:6 (HNO_3_:H_2_SO_4_) was due to the ability of excess sulfuric acid to reduce the oxidative properties of the mixture. The nitration process proceeds smoothly and in good yield (65%). No oxidation of the amino group was observed; thus, this protocol eliminates the additional steps of the introduction and removal of protecting groups. As a result, 6-nitrotetrazolo[1,5-*a*]pyrimidine-7-amine **4** was obtained in 65% yield (Scheme 2).

The structure of compound **4** was established based on ^1^H, ^13^C NMR spectroscopy, IR spectroscopy (Figures S2 and S6, see Supplementary Materials), and elemental analysis data. The ^1^H NMR spectrum of compound **4** shows a single-proton signal in the region of δ 9.01 ppm. Corresponding to the resonance of the 5-H atom of the CH group, two single-proton singlet signals correspond to the protons of the amino group in the regions δ 8.89 ppm and 8.27 ppm.

In the IR spectrum of compound **4**, the valence vibration of the azide fragment can be observed, which corresponds to an intense band in the region of 2137 cm^−1^. This shows that 6-nitrotetrazolo[1,5-*a*]pyrimidine-7-amine **4** is in an azide form in the solid state. The absorption band in the region of 3436 cm^−1^ corresponds to the amino group. In the region of 1633 cm^−1^, 1329 cm^−1^ is the absorption band of nitro group.

It is known that the nitro group can act both as a leaving group and as a cryptoform of the amino group [17]. Thus, in the case of 6-nitroazolo[1,5-*a*]pyrimidines containing an amino group at the 5 or 7 position, it appears possible to complete the heterocyclic system to the corresponding purine. Therefore, the next stage of the study was the development of methods for the obtaining of pyrimidine-2,4,5-triamine **6**. In our previous work, derivatives of (phenyldiazenyl)tetrazolo[1,5-*a*]pyrimidine-7-amine [16] were prepared by condensation of tetrazolo[1,5-*a*]pyrimidine-7-amine **3** with 2-oxo-N-phenylacetohydrazonoyl cyanide derivatives. As an alternative approach for the preparation of vicinal diamino derivatives of tetrazolopyrimidines, several attempts were made to reduce the (phenyldiazenyl)tetrazolo[1,5-*a*]pyrimidine-7-amine, which contains an azo fragment as a cryptoform of the amino group that can be obtained by reductive cleavage. However, we tried different reducing agents such as sodium dithionite, lithium aluminohydride, and zinc in acetic acid, but the initial azo compound was isolated from the reaction mass.

One of the synthetic strategies for obtaining 2-aminopurine is the synthesis of a tetrazolo[1,5-*a*]pyrimidine-6,7-diamine. To obtain it, it is necessary to reduce tetrazolo[1,5-*a*]pyrimidine-7-amine **3**, and this must be completed selectively. This heterocycle has two reaction centers capable of reduction: the nitro group and the tetrazole ring. However, we did not succeed in the selective reduction process of the nitro group without affecting the tetrazole cycle. We can assume that the tetrazole fragment is more reactive to reduction compared to the nitro group, which is located in the sixth position. The following reducing agents were used for this purpose: attempts to use zinc in acetic acid and in ethanol with ammonia did not lead to the formation of the desired intermediate. The typical reducing agent of iron and hydrochloric acid as a solvent failed to isolate 5-nitropyrimidine-2,4-diamine **5**. Sodium dithionite, which played the role of a reducing agent in the next step to obtain triaminopyrimidine, was ineffective in the selective reduction of tetrazole. In addition, H_2_/Pd, which is often used for the reduction of nitro groups, failed to yield an intermediate. In some cases, nothing happened, while in others, the tetrazole was reduced but the nitro group remained unreduced.

The construction of 5-nitropyrimidine-2,4-diamine **5** based on aminotetrazole is novel in the synthesis of nitrogenous heterocycles [18]. Thus, we have developed the following approach for the synthesis of pyrimidine-2,4,5-triamine **6**: the reduction of nitrotetrazolo[1,5-*a*]pyrimidin-7-amine **4**.

**Scheme 3:**
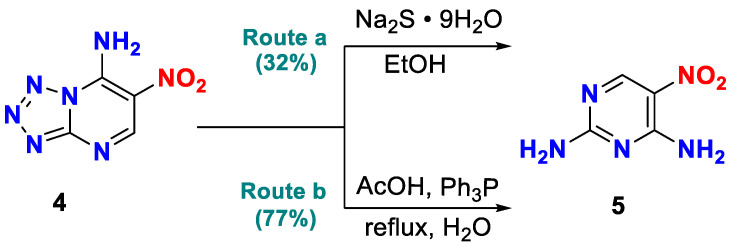
Synthesis of 5-nitropyrimidine-2,4-diamine **5**.

It turned out that sodium sulfide nonahydrate, aqueous in alcoholic medium, undergoes a reduction of the tetrazole fragment to the amino group, which leads to the preparation of 5-nitropyrimidine-2,4-diamine **5** (32%) (Scheme 3. Route a).

A selection of conditions was required to make the yields of the compounds preparative. The conditions were selected to make the yields of the target product preparative. The results obtained clearly demonstrated that the best activating reagent was triphenylphosphine (Scheme 3. Route b) when used in an amount of 1.0, equivalent in acetic acid for 2 h, leads to 5-nitropyrimidine-2,4-diamine **5** (77%) (Table 1, entry 4).

**Figure 2:**
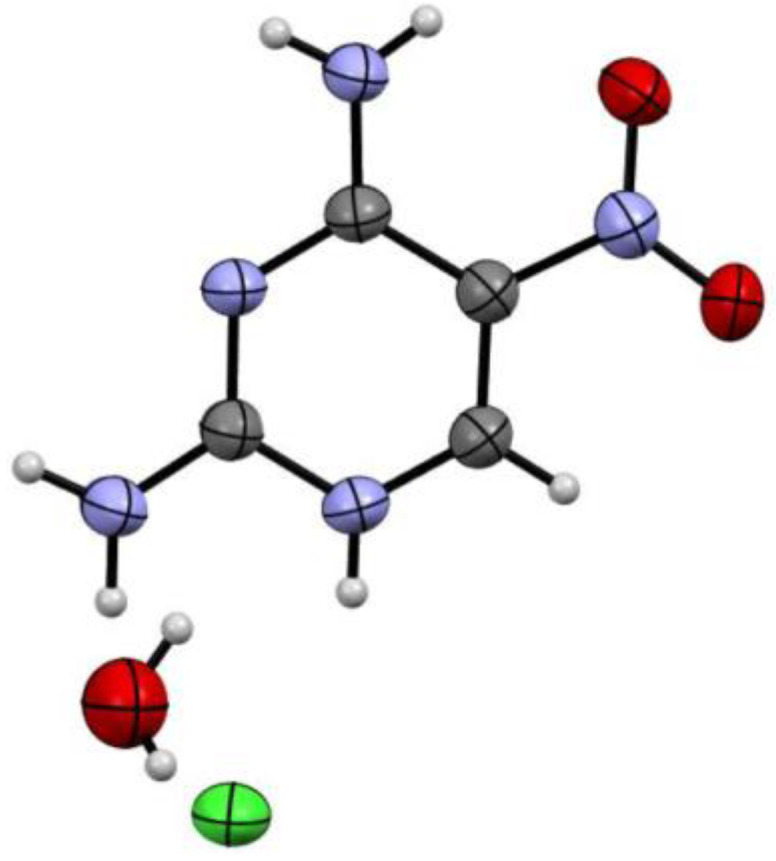
X-ray diffraction analysis of compound 5-nitropyrimidine-2,4-diamine **5**.

The ^1^H spectrum of 5-nitropyrimidine-2,4-diamine **5** shows a single-proton signal in the region of 8.84 ppm corresponding to the resonance of the 6-H-atom of the pyrimidine fragment and two two-proton doublets corresponding to the protons of the amino group (δ = 7.98 ppm; 7.45 ppm) (Figure S3, see Supplementary Materials). In addition, the structure of 5-nitropyrimidine-2,4-diamine **5** was finally determined by X-ray diffraction analysis (Figure 2).

Nitro compounds are important building blocks for further important transformations [19].

**Scheme 4:**
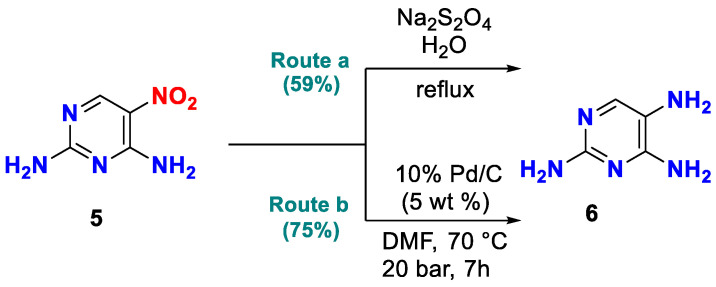
Synthesis of pyrimidine-2,4,5-triamine **6**.

In the next step, we attempted the reduction of the nitro group to obtain the target pyrimidine-2,4,5-triamine **6**. In the next step, we attempted the reduction of the nitro group to obtain the target pyrimidine-2,4,5-triamine **6**. It is worth noting that reduction by tin or iron in an acidic medium leads to oil formation in the reaction mass (Table 2, entry 2,3). At the same time, in contrast to the reduction of 6-nitrotetrazolo[1,5-*a*]pyrimidin-7-amine **4**, the use of sodium dithionite favors the reduction of the nitro group to the corresponding amine (Scheme 4. Route a), so pyrimidine-2,4,5-triamine **6** was obtained in 59% yield (Table 2, entry 1). Apparently, the energy barrier of this reaction is lower for pyrimidine than for its azoloannelated derivative. Nevertheless, the most successful result in this reaction was the reduction by hydrogen on palladium in dimethylformamide under heating at 20 bar, which gave the desired pyrimidine-2,4,5-triamine **6** in 75% yield (Scheme 4. Route b).

The structure of compound **6** was justified by ^1^H, ^13^C NMR spectroscopy, IR spectroscopy, and elemental analysis data. The ^1^H NMR spectrum of compound **6** shows a single-proton signal in the region of δ 7.22 ppm. Corresponding to the resonance of the 6-H atom of the CH group, three single-proton singlet signals correspond to the protons of the amino group in the regions of δ 5.86 ppm, 4.95 ppm, and 3.59 ppm. The presence of the amino groups in the IR spectrum is also confirmed by the presence of a broadened band in the region of 3000–3400 cm^−1^ (Figures S4 and S8, see Supplementary Materials).

The final step of the 2-aminopurine synthesis is the construction of the imidazole cycle. Despite the simplicity of the reaction, different derivatives of vicinal diamines may require different conditions [9,10,11,12,13,14].

**Scheme 5:**
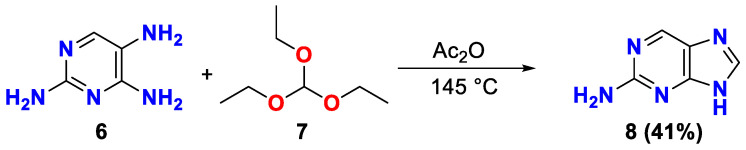
Synthesis of 2-aminopurine **8.**

Traube synthesis is a classic method to obtain purines through the annulation of the imidazole fragment into ortho-diaminopyrimidines [20]. This method was not applicable to us. For the final cyclization of pyrimidine-2,4,5-triamine **6** to 2-aminopurine **8**, we used different formylation reagents (Table 3). The use of triethylorthoformate, acetic acid as a catalyst, and N-formylmorpholine as a formylation agent in formic acid did not lead to the preparation of the target product **8**. The results obtained clearly demonstrated that the best reagents were triethylorthoformate and acetic anhydride (Scheme 5), and 2-aminopurine **8** was obtained with a yield of 41%.

### 2.2. Crystallography

**Figure 3:**
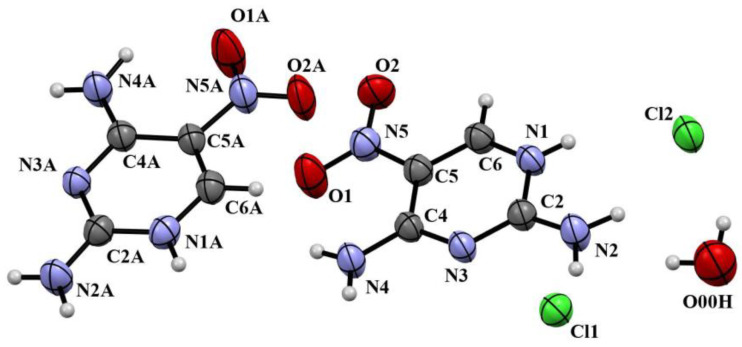
The compound in the thermal ellipsoid of the 50% probability level.

According to the XRD data, two independent molecules of the compound are crystallized with a molecule of H_2_O in the centrosymmetric space group of the triclinic system. In the result, the structurally independent unit C_4_H_7_ClN_5_O_2.5_ was used for all calculations. The geometry of the independent heterocyclic molecules has an insignificant distinction; however, all bond distances and angles are near expectations. Both molecules are planar, with insignificant dihedral angles between the NO_2_ group and the plane of the heterocycle. The general geometry of the molecules is shown in Figure 3. The crystal packing is ordered by the strong system of the H-bonds (Table S1, Supplementary Materials).

## 3. Materials and Methods

### 3.1. Chemical Experiment

Commercial reagents were obtained from Sigma-Aldrich, Acros Organics, or Alfa Aesar and used without any preprocessing. All workup and purification procedures were carried out using analytical-grade solvents. One-dimensional ^1^H- and ^13^C-NMR spectra were acquired on a Bruker DRX-400 instrument (Karlsruhe, Germany) (400 and 101 MHz, respectively) or a Bruker Avance NEO 600 instrument (151 MHz), utilizing DMSO-d6 as solvents and an external reference, respectively. Chemical shifts are expressed in δ (parts per million, ppm) values, and coupling constants are expressed in hertz (Hz). The following abbreviations are used for the multiplicity of NMR signals: s, singlet; d, doublet. IR spectra were recorded on a Bruker α spectrometer equipped with a ZnSe ATR accessory. Elemental analysis was performed on a PerkinElmer PE 2400 elemental analyzer (Waltham, MA, USA). Melting points were determined on a Stuart SMP3 (Staffordshire, UK) and are uncorrected. The monitoring of the reaction progress was performed using TLC on Sorbfil plates (Imid LTD, Krasnodar, Russia) (the eluent is EtOAc).

The procedure for the synthesis of tetrazolo[1,5-*a*]pyrimidine-7-amine (**3**) was as follows: To a solution of 1H-tetrazol-5-amine **1** (0.01 mol, 1 eq.) in an equimolar mixture of pyridine (10 mL)/acetic acid (14 mL) was added morpholineacrylonitrile **2** (0.01 mol, 1 eq.). The resulting mixture was stirred at 135 °C for 4 h. The resulting precipitate was filtered off, washed with 20 mL EtOH, and dried in air. Beige powder (yield 71%). m. p. 274–276 °C. FT-IR (neat) ν_max_ (cm^−1^): 3108, 3287. ^1^H-NMR (400 MHz, DMSO-d6) δ (ppm) 6.58 (1H, d, J = 7.4 Hz, 5-H), 7.76 (2H, s, NH_2_), 8.92 (1H, d, J = 7.4, 5-H). ^13^C-NMR (100 MHz, DMSO-d6) δ (ppm) 104.3, 132.8, 155.5, 162.2 Calcd for C_4_H_4_N_6_: C 35.30, H 2.96, N, 61.74; found: C 35.37, H 2.91, N 61.72.

The procedure for the synthesis of 6-nitrotetetrazolo[1,5-*a*]pyrimidine-7-amine (**4**) was as follows: To 3.75 mL (0.09 mol) of concentrated nitric acid cooled to 0–5 °C, concentrated sulfuric acid 28.94 mL (0.54 mol) was added. To the resulting mixture, tetrazolo[1,5-*a*]pyrimidine-7-amine **3** was added in small portions. The reaction mixture was heated for 4 h at 80 °C, cooled to room temperature, poured on ice, and neutralized with aqueous ammonia solution (25%) (80 mL) to pH = 7. The resulting precipitate was filtered off and washed thoroughly with water (40 mL). White powder (yield 65%). m. p. 233–235 °C. FT-IR (neat) ν_max_ (cm^−1^): 1329, 1633, 2137, 3436. ^1^H-NMR (400 MHz, DMSO-d6) δ (ppm) 8.27 (1H, s, NH_2_), 8.89 (1H, s, NH_2_), 9.01 (1H, s, 5-H). ^13^C-NMR (100 MHz, DMSO-d6) δ (ppm) 124.3, 157.3, 157.9, 163.8 Calcd for C_4_H_3_N_7_O_2_: C 26.53, H 1.67, N, 54.14; found: C 26.44, H 1.75, N 54.09.

The procedure for the synthesis of 5-nitropyrimidine-2,4-diamine (**5a**) was as follows: To 6-nitrotetrazolo[1,5-*a*]pyrimidin-7-amine **4** (4.9 mmol) was added 10 mL of ethyl alcohol. Sodium sulfide (4.9 mmol) was added to the resulting mixture in small portions at 60 °C and stirred, and then it was cooled at room temperature. The resulting precipitate was filtered off and washed thoroughly with water (20 mL).

The procedure for the synthesis of 5-nitropyrimidine-2,4-diamine (**5b**) was as follows: To acetic acid (10 mL) was added 6-nitrotetrazolo[1,5-*a*]pyrimidin-7-amine **4** (2.8 mmol). The reaction mixture was stirred at 130 °C, and upon reaching this temperature, triphenylphosphine (2.8 mmol) was added to the mixture in small portions and endured for one hour. Then 0.1 mL of water was added, and the mixture cooled after one hour; the obtained precipitate was filtered and washed with 20 mL of ethyl alcohol. Pale yellow powder (yield 77%). m. p. > 300 °C. FT-IR (neat) ν_max_ (cm^−1^): 1375, 1625, 3417. ^1^H-NMR (400 MHz, DMSO-d6) δ (ppm) 7.46 (2H, d, J = 56 Hz, 5-H), 7.97 (2H, d, J = 32 Hz, 1-H), 8.84 (1H, s, 6-H). ^13^C-NMR (100 MHz, DMSO-d6) δ (ppm) 120.1, 157.9, 158.5, 163.9 Calcd for C_4_H_5_N_5_O_2_: C 30.95, H 3.22, N, 45.20; found: C 30.98, H 3.31, N 45.07.

The procedure for the synthesis of pyrimidine-2,4,5-triamine (**6a**) was as follows: To 5-nitropyrimidine-2,4-diamine **5** (3.6 mmol) was added 10 mL of water, stirred at 80 °C; sodium dithionite (7.2 mmol) was added for 3–5 min and then stirred at 60 °C, after which sodium carbonate (0.018 mol) was added. The reaction mixture was evaporated at a rotary evaporator, crushed, and extracted with isopropyl alcohol (30 mL). The resulting precipitate was filtered off (the procedure was repeated 3 times in total). The filtrate was evaporated at the rotary evaporator and dried in air.

The procedure for the synthesis of pyrimidine-2,4,5-triamine (**6b**) is as follows: A mixture of the corresponding pyrimidine-2,4,5-triamine 6 (1 mmol) and 10% (by weight) Pd/C (5 wt %) in DMF (5 mL) was hydrogenated in an autoclave at 70 °C and 20 bar hydrogen pressure for 5–7 h. The resulting solution was filtered off the Pd/C, the solvent was removed under reduced pressure, and the crude oil was crystalized in 15 mL isopropyl alcohol; the precipitate was filtered and dried in air. Red-brown solid (yield 75%). m. p. 248–250 °C. FT-IR (neat) ν_max_ (cm^−1^): 1329, 1359, 1563, 3271. ^1^H-NMR (400 MHz, DMSO-d6) δ (ppm) 3.59 (1H, s, NH_2_), 4.95 (1H, s, NH_2_), 5.86 (1H, s, NH_2_), 7.22 (1H, s, 6-H). ^13^C-NMR (151 MHz, DMF-d_7_) δ (ppm) 118.7, 140.9, 156.6, 158.1 Calcd for C_4_H_7_N_5_: C 38.39, H 5.64, N, 55.97; found: C 38.31, H 5.60, N 56.09.

The procedure for the synthesis of 2-aminopurine (**8**) is as follows: To pyrimidine-2,4,5-triamine **6** (0.4 mmol) was added triethylorthoformate 3.0 mL (0.018 mol) and acetic anhydride 1.0 mL (0.01 mol) boiled at 145 °C for 1.5 h; the reaction mixture was cooled and evaporated. A solution of NaOH (0.02 mol) in 5 mL of water was added and boiled for 10 min; the resulting solution was cooled, and hydrochloric acid 35% 1.6 mL (0.02 mol) was added; the pH was brought to neutral with 10 mL of CH_3_COONa 4M. The resulting solution was evaporated at the evaporator and then 15 mL acetone was added; the precipitate formed was filtered off and the filtrate was evaporated at the evaporator. The product was purified by flash chromatography with a mixture of CHCl_3_ and MeOH (8:2). Pale yellow powder (yield 41%). m. p. 280–282 °C. FT-IR (neat) ν_max_ (cm^−1^): 1614, 1663, 3215. ^1^H-NMR (400 MHz, DMSO-d6) δ (ppm) 6.29 (2H, s, NH_2_), 8.03 (1H, s, 8-H), 8.56 (1H, s, 6-H). ^13^C-NMR (100 MHz, DMSO-d6) δ (ppm) 125.4, 144.1, 156.6, 158.4 Calcd for C_5_H_5_N_5_: C 44.44, H 3.73, N, 51.83; found: C 44.32, H 3.82, N 51.86.

### 3.2. Crystallography Experiment

The XRD analyses were carried out using equipment from the Center for Joint Use “Spectroscopy and Analysis of Organic Compounds” at the Postovsky Institute of Organic Synthesis of the Russian Academy of Sciences (Ural Branch). The experiment was accomplished on the automated X-ray diffractometer “Xcalibur 3” with a CCD detector on the standard procedure (MoKα-irradiation, graphite monochromator, ω-scans with 1o step at T = 295(2) K). Empirical absorption correction was applied. The solution and refinement of the structures were accomplished using the Olex program package [21]. The structures were solved by the method of the intrinsic phases in the ShelXT program and refined by ShelXL by a full-matrix least-squared method for non-hydrogen atoms [22]. The H-atoms at C-H bonds were placed in the calculated positions; the H-atoms of the O-H and N-H bonds were solved by a direct method and were refined independently in isotropic approximation.

Crystal data for C_4_H_7_ClN_5_O_2.5_ (M = 200.60 g/mol): triclinic, space group P-1, a = 5.5123(3) Å, b = 9.8766(5) Å, c = 15.7380(10) Å, α = 73.518(5)°, β = 88.104(5)°, γ = 86.787(4)°, V = 820.19(8) Å3, Z = 4, T = 295(2) K, μ(MoKα) = 0.443 mm^−1^, Dcalc = 1.624 g/cm^3^, 8459 reflections measured (7.406° ≤ 2Θ ≤ 60.94°), 4484 unique [Rint = 0.0451, Rsigma = 0.0649], which were used in all calculations. The final R1 = 0.0600, wR2 = 0.1431 (I > 2σ(I)), and R1 = 0.0955, wR2 = 0.1835 (all data). GooF = 1.041. Largest diff. peak/hole 0.41/−0.50.

The XRD data were deposited in the Cambridge Structural Database with the number CCDC 2308999. These data can be requested free of charge via www.ccdc.cam.ac.uk (accessed on 20 November 2023).

## 4. Conclusions

A new method for the preparation of 2-aminopurine, as an important compound, has been established, and an important conceptual synthetic principle has been utilized. The method includes cyclization, nitro-formation (functionalization), and reduction with the destruction of the initial heterocycle. We propose the synthetic sequence of targeted final cyclization as a reconstructive methodology in the synthesis of heterocycles

## Figures and Tables

**Table 1:** Optimization for the preparation to 5-nitropyrimidine-2,4-diamine **5** ^1^.

No.	Solvent ^2^	Reducing Agent	X, Equiv	Reaction Condition ^3^	Yield, % ^5^
entry 1	EtOH	Na_2_S•9H_2_O	1.0	60 °C, 0.1 h	32%
entry 2	HCl	Fe	8.0	60 °C, 2 h	-
entry 3	AcOH	Zn	10.0	reflux, 2 h	-
entry 4	AcOH	Ph_3_P	1.0	reflux, 2 h	77%
entry 5	AcOH	Cu	1.0	reflux, 0.2 h	-
entry 6	THF	Ph_3_P	1.0	reflux, 2 h	-
entry 7	H_2_O	Na_2_S_2_O_4_	8.0	reflux, 0.2 h	-
entry 8	NH_4_OH, EtOH	Zn	4.0	70 °C, 1 h	-
entry 9	EtOH	^6^ H_2_/Pd	-	^4^ 50 °C, 4 h	-

^1^ Reaction conditions: **4** (4.9 mmol); ^2^ amount of solvent—10 mL; ^3^ conventional heating with an oil bath; ^4^ autoclave; ^5^ isolated yield; and ^6^ 10% Pd/C (5 wt %).

**Table 2:** Optimization for the preparation of pyrimidine-2,4,5-triamine **6** ^1^.

No.	Solvent ^2^	Reducing Agent	X, Equiv	Reaction Condition ^3^	Yield, % ^5^
entry 1	H_2_O	Na_2_S_2_O_4_	6.0	reflux, 0.2 h	59
entry 2	TEOF, AcOH	Fe	10.0	130 °C, 5 h	-
entry 3	HCl	Sn	2.0	110 °C, 1 h	-
entry 4	DMF	^6^ H_2_/Pd	0.05	^4^ 100 °C, 4 h	75

^1^ Reaction conditions: **5** (4.9 mmol); ^2^ amount of solvent—10 mL; ^3^ conventional heating with an oil bath; ^4^ autoclave; ^5^ isolated yield; and ^6^ 10% Pd/C (5 wt %).

**Table 3:** Optimization for the preparation of 2-aminopurine **8** ^1^.

No.	Solvent ^2^	Formylation Agent	mL, Qty/Equiv	Reaction Condition ^3^	Yield, % ^4^
entry 1	-	HCOOH	6.0/45.0	reflux, 3 h	-
entry 2	-	TEOF	6.0/45.0	reflux, 3h	-
entry 3	AcOH	TEOF	1.0/3.0	reflux, 3 h	-
entry 4	AcOH	TEOF	1.0/5.0	reflux, 3 h	-
entry 5	N-Formylmorpholine	HCOOH	1.0/6.5	reflux, 3 h	-
entry 6	Ac_2_O	TEOF	3.0/25.0	reflux, 2 h	41

^1^ Reaction conditions: **6** (0.4 mmol); ^2^ amount of solvent—3 mL; ^3^ conventional heating with an oil bath; and ^4^ isolated yield.

## Data Availability

The data presented in this study are available in article and Supplementary Materials.

## Supplementary Materials

The following supporting information can be downloaded at: https://www.mdpi.com/article/10.3390/molecules29010134/s1, Figures S1–S4: ^1^H- and ^13^C-NMR spectra of compounds **3**–**6**; Figures S5–S8: IR spectra of compounds **3**–**6**. Table S1: Hydrogen bonds with H.A < r(A) + 2.000 Å and <DHA> 110°.
