# Supplementary material for: Reconstructive Methodology in the Synthesis of 2-Aminopurine
Source: Molecules. 2023 Dec 25;29(1):134. doi: 10.3390/molecules29010134 (PMC10780053; doi:10.3390/molecules29010134)
Supplement: Supplementary file 1 [file molecules-29-00134-s001.zip › molecules-2772867-supplementary.pdf]

Table of contents

1. Spectra of compounds 2
2. Crystallography 8

1. Spectra of compounds

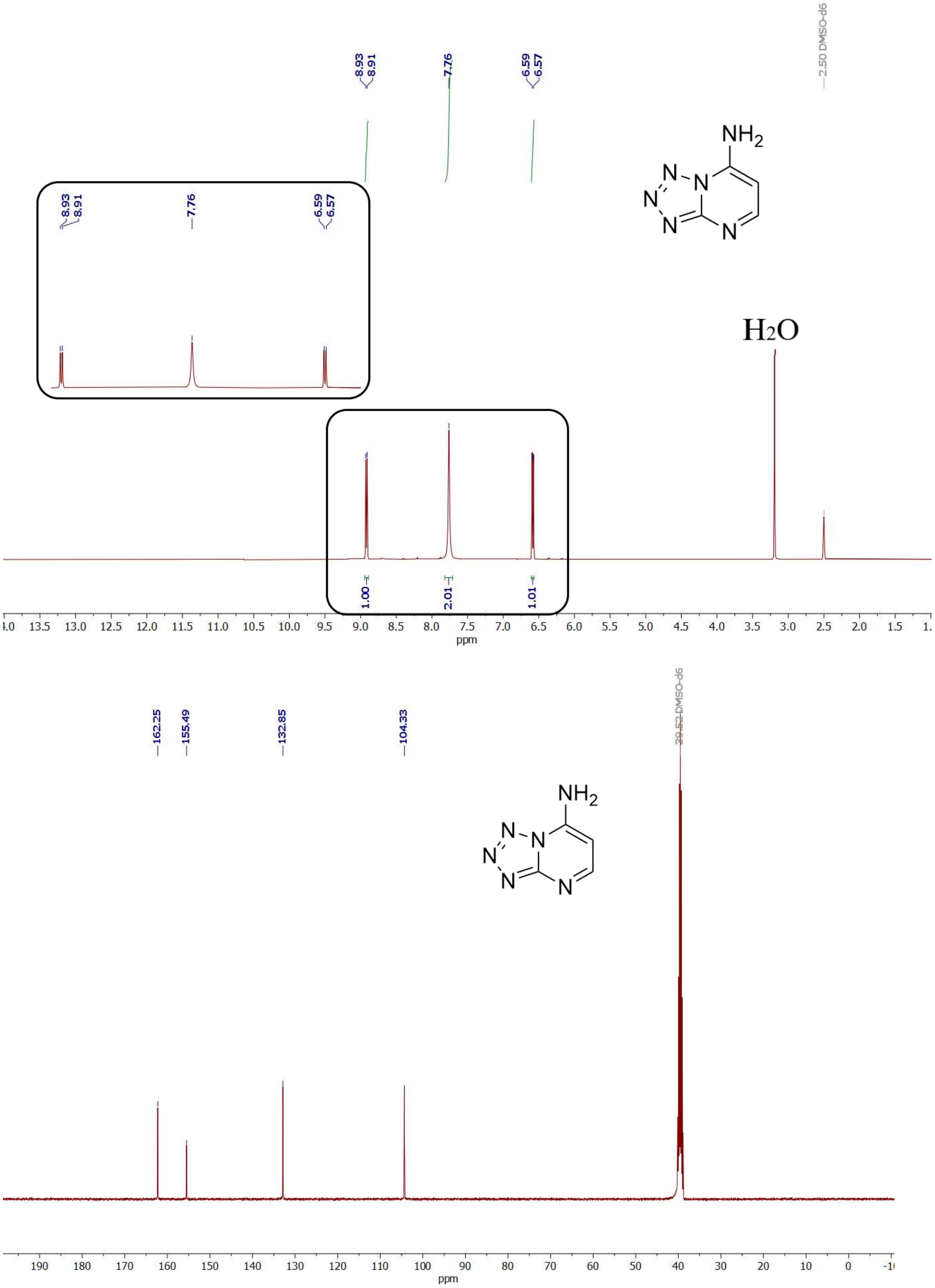

Figure S1. ^{1}H NMR (400 MHz, DMSO-d6) and ^{13}C NMR (100 MHz, DMSO-d6) spectra of tetrazolo[1,5-*a*]pyrimidin-7-amine **3**

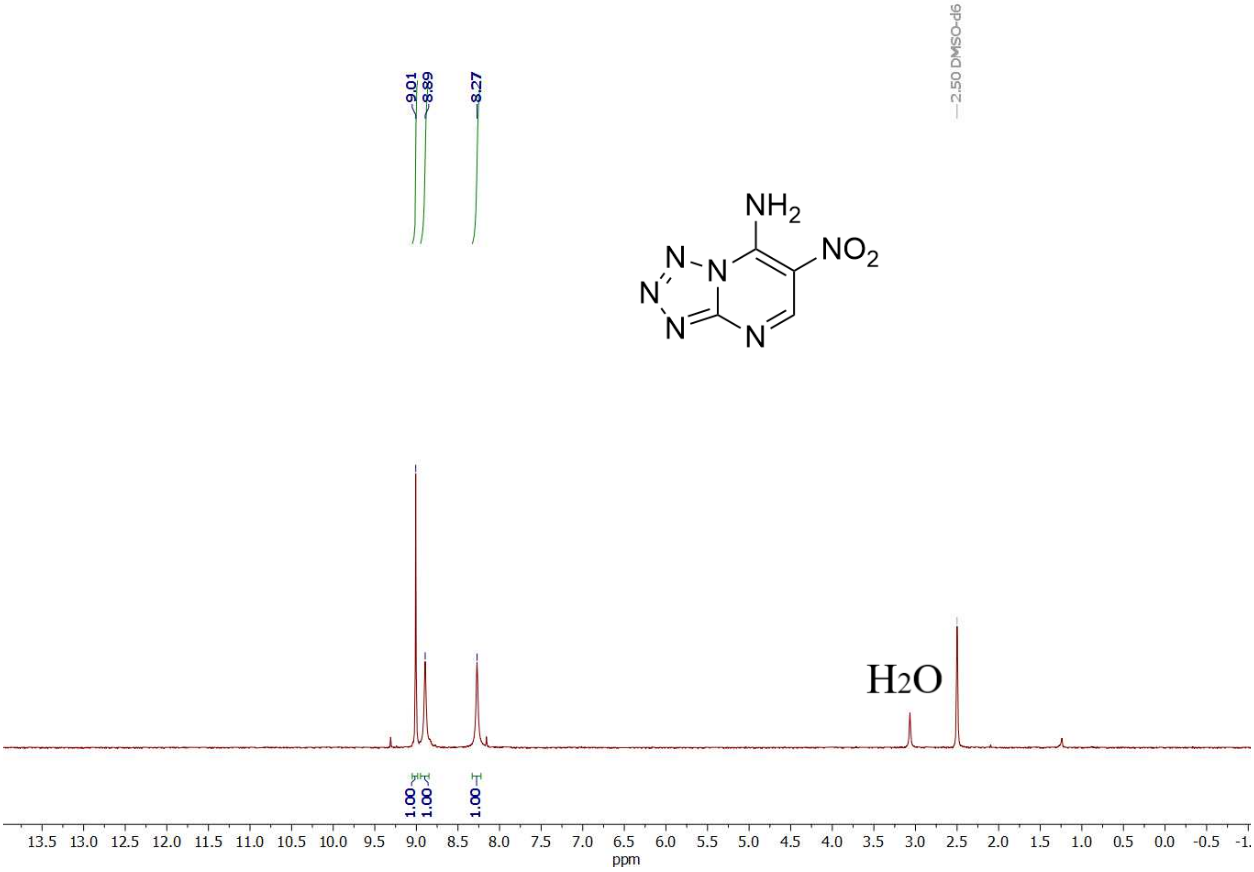

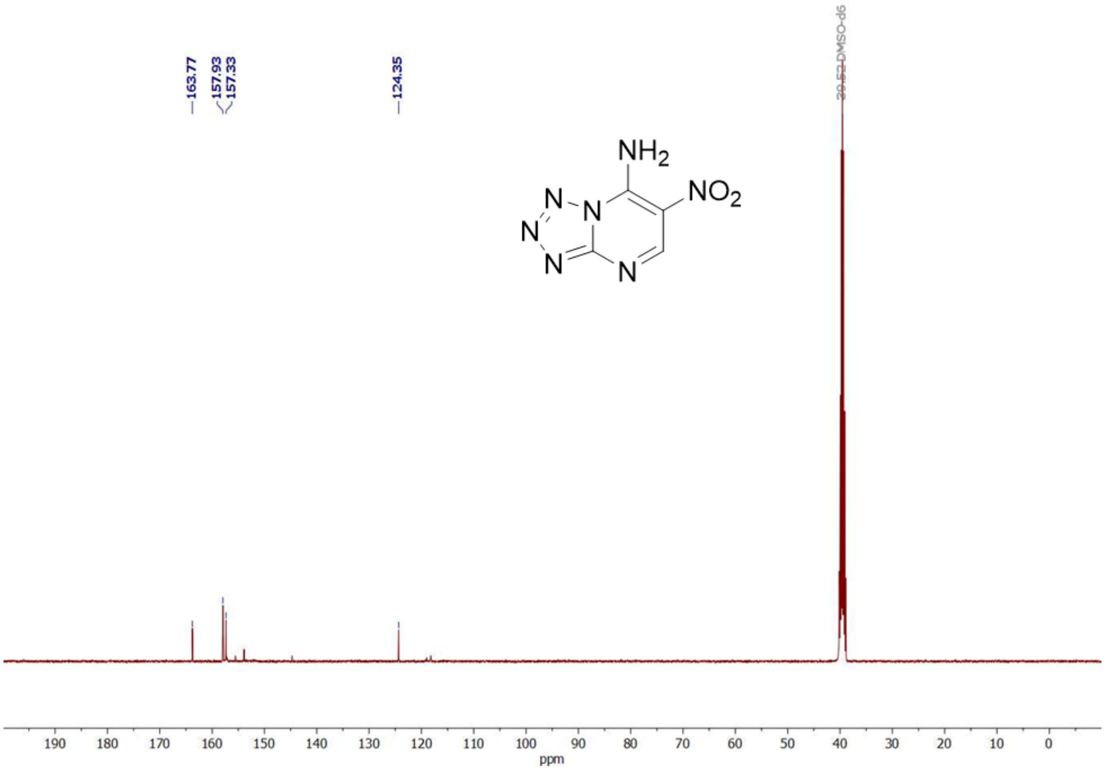

Figure S2. ^{1}H NMR (400 MHz, DMSO-d6) and ^{13}C NMR (100 MHz, DMSO-d6) spectra of 6-nitrotetrazolo[1,5-*a*]pyrimidin-7-amine **4**

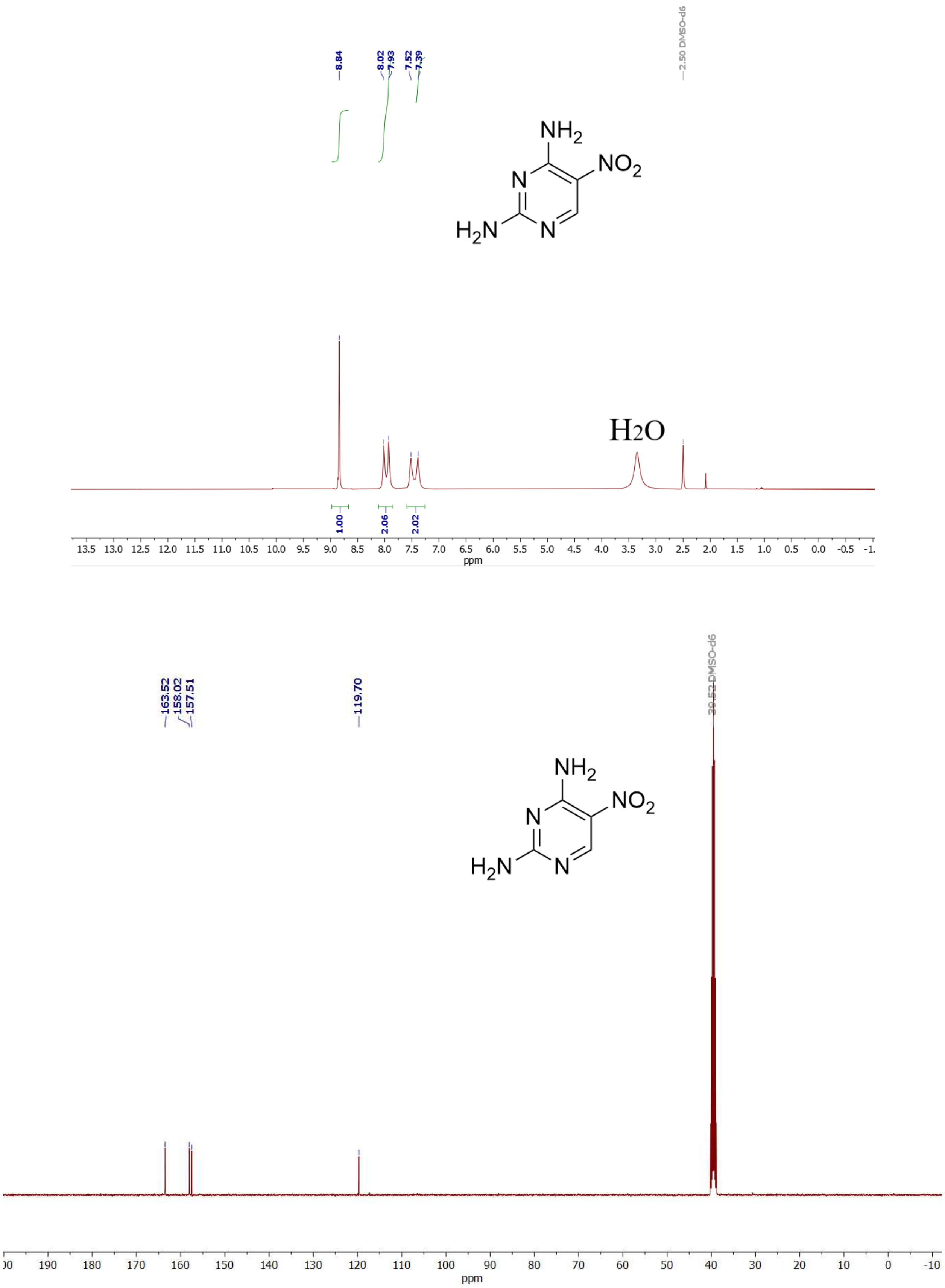

Figure S3. ^{1}H NMR (400 MHz, DMSO-d6) and ^{13}C NMR (100 MHz, DMSO-d6) spectra of **5**

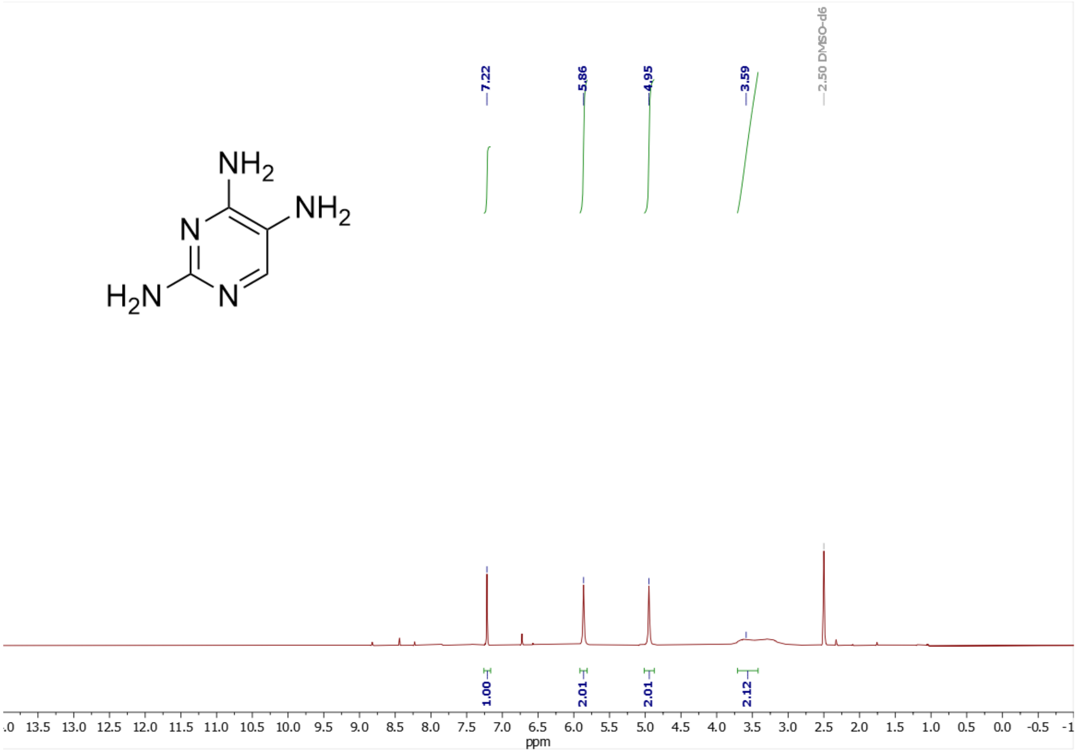

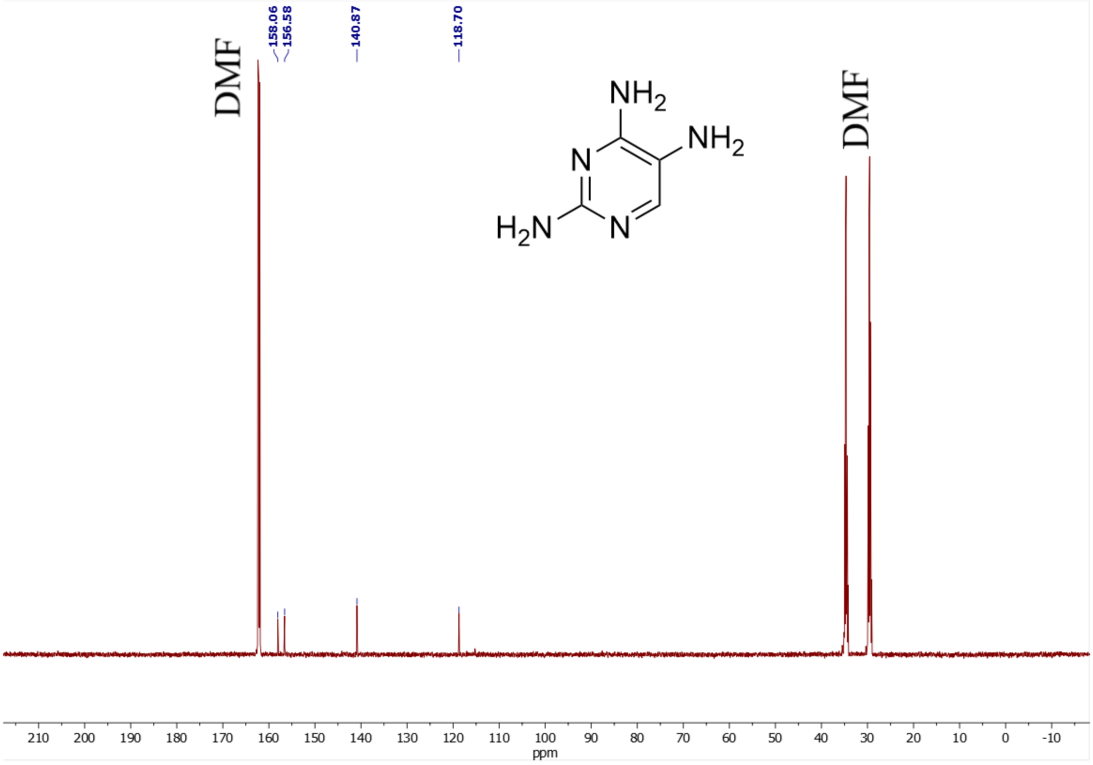

Figure S4. ^{1}H NMR (400 MHz, DMSO-d6) and ^{13}C NMR (151 MHz, DMF-d7) spectra of pyrimidine-2,4,5-triamine **6**

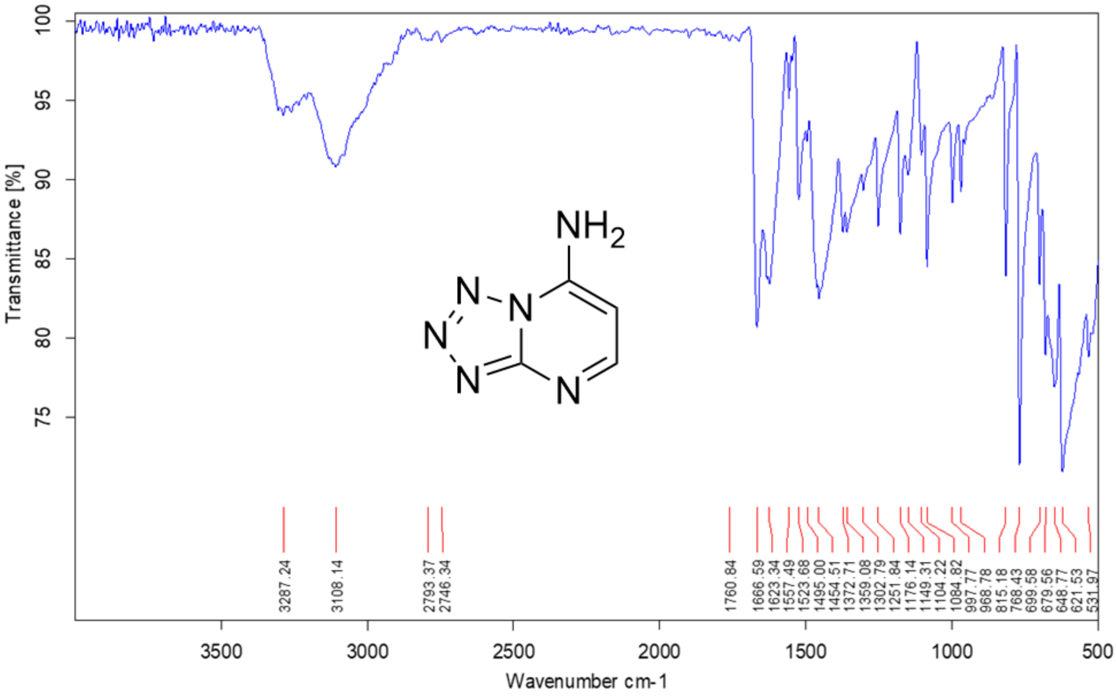

Figure S5. IR spectra of tetrazolo[1,5-*a*]pyrimidin-7-amine **3**

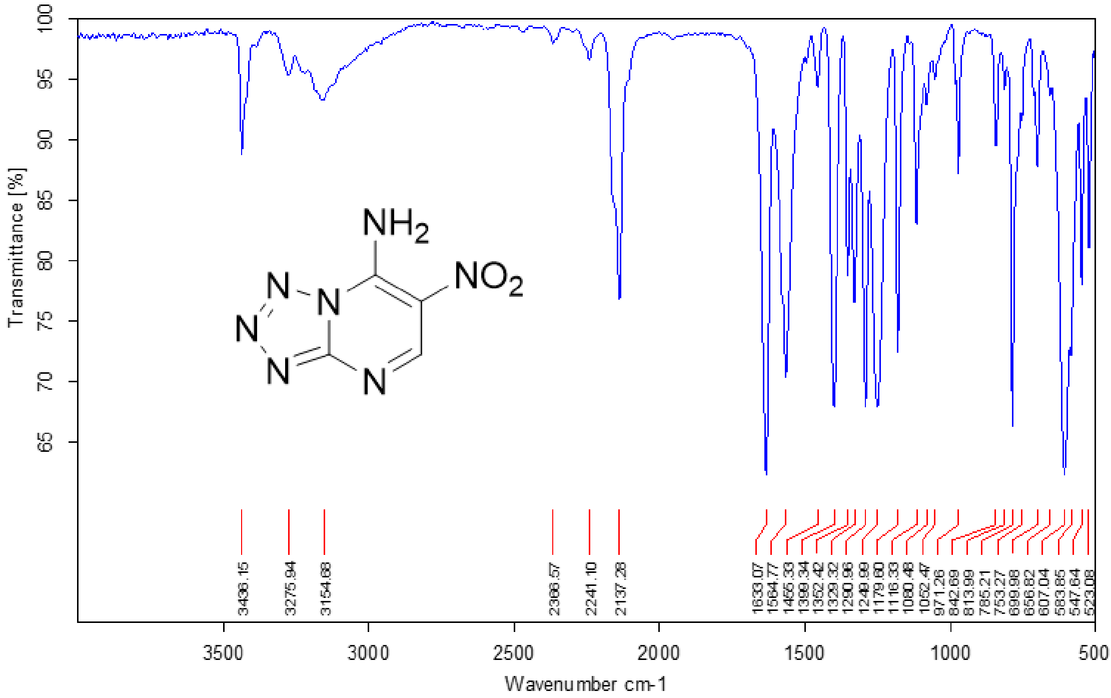

Figure S6. IR spectra of 6-nitrotetrazolo[1,5-*a*]pyrimidin-7-amine **4**

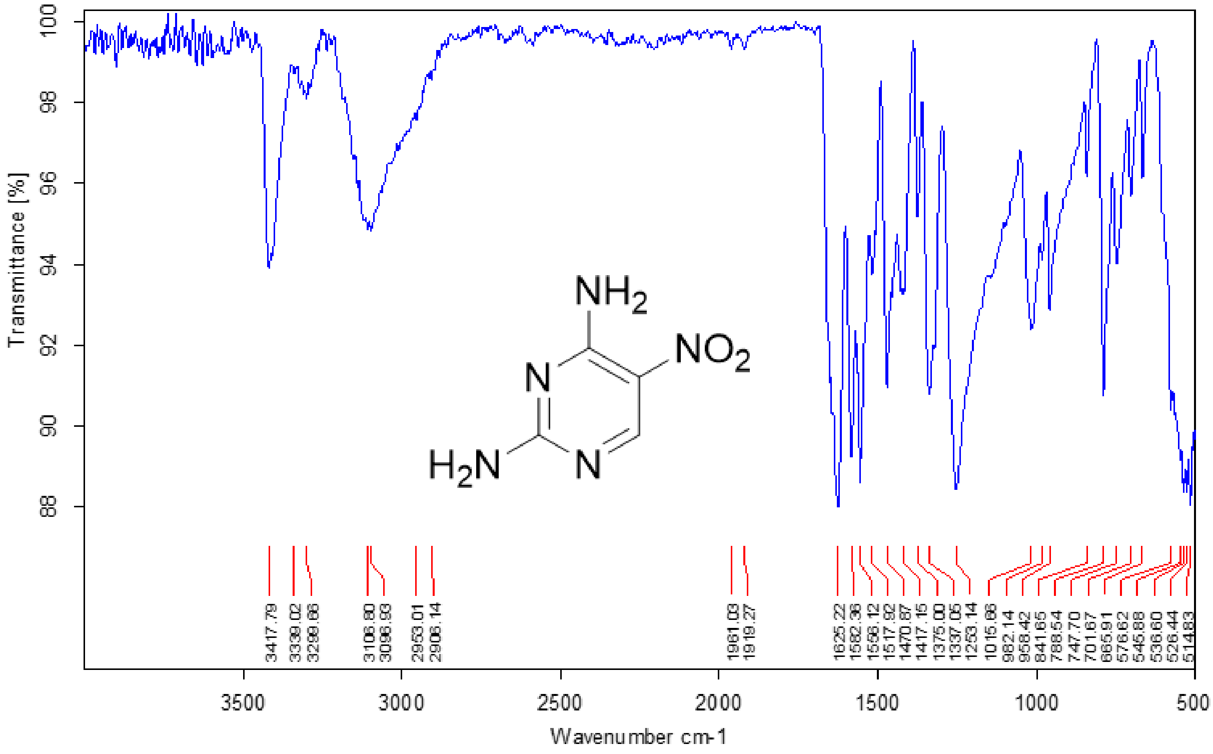

Figure S7. IR spectra of 5-nitropyrimidine-2,4-diamine **5**

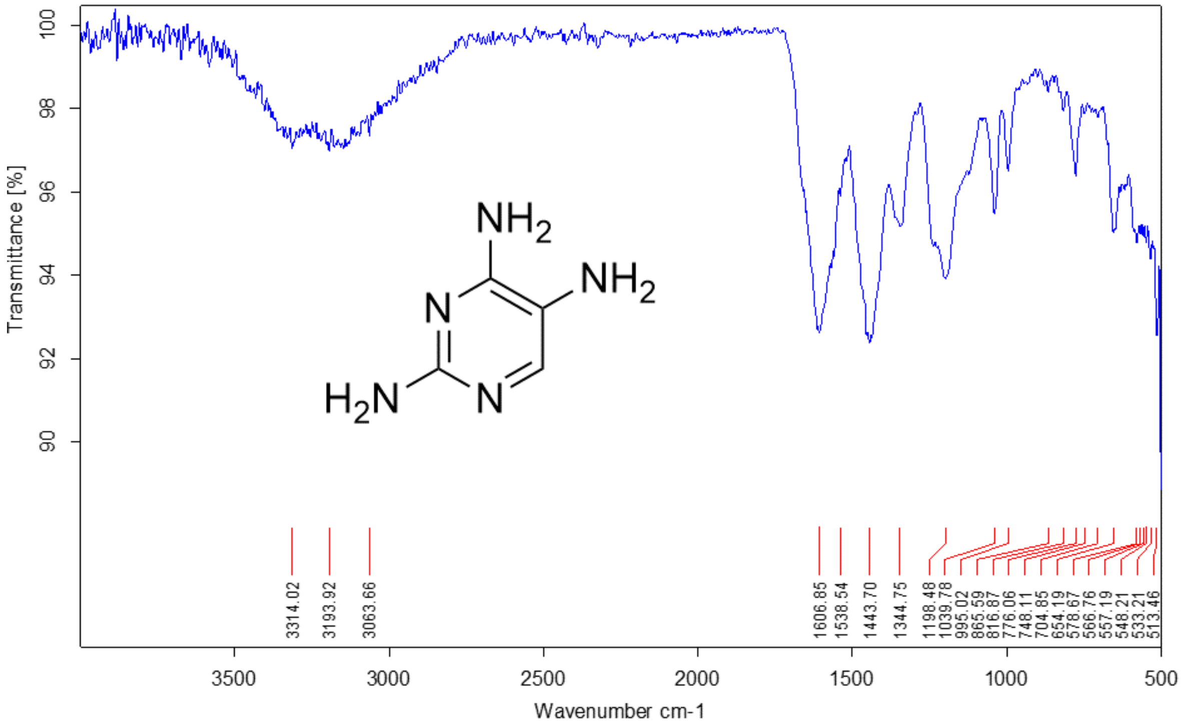

Figure S8. IR spectra of pyrimidine-2,4,5-triamine **6**

2. Crystallography

Table S1. Hydrogen bonds with H..A < r(A) + 2.000 Å and <DHA > 110º.

D-H	d(D-H)	d(H..A)	<DHA	d(D..A)	A
N1A-H1A	0.86(3)	2.26(3)	163(2)	3.093(4)	Cl1 [2-x, 1-y, 1-z]
N2A-H2AA	0.95(4)	1.87(4)	176(2)	2.814(4)	O00H [2-x, 1-y, 1-z]
N2A-H2AB	0.85(4)	2.43(4)	159(2)	3.237(4)	Cl2 [x, y+1, z-1]
N4A-H4AA	0.95(4)	2.48(4)	146(2)	3.312(4)	Cl2 [1-x, -y, 1-z]
N4A-H4AA	0.95(4)	2.02(4)	122(2)	2.658(4)	O1A
N4A-H4AB	0.80(4)	2.28(4)	167(2)	3.064(4)	N3A [1-x, 1-y, -z]
N1-H1	0.81(3)	2.38(3)	154(2)	3.129(4)	Cl2
N1-H1	0.81(3)	2.58(3)	124(2)	3.100(4)	O1A [1-x, -y, 1-z]
N2-H2A	0.87(4)	2.33(4)	166(2)	3.176(4)	Cl1 [x+1, y, z]
N2-H2B	0.92(4)	2.38(4)	151(2)	3.212(4)	Cl2
N4-H4A	0.82(3)	2.57(3)	146(2)	3.287(4)	Cl1 [1-x, 1-y, 1-z]
N4-H4A	0.82(3)	2.12(3)	125(2)	2.684(4)	O1
N4-H4B	0.85(3)	2.22(3)	169(2)	3.064(4)	N3 [2-x, 1-y, 1-z]
C6-H6	0.930	2.414	125.3	3.047(4)	O1A [1-x, -y, 1-z]
C6A-H6A	0.930	2.390	163.4	3.292(4)	O1 [x+1, y, z]
